# Detection of AI-2 Receptors in Genomes of *Enterobacteriaceae* Suggests a Role of Type-2 Quorum Sensing in Closed Ecosystems

**DOI:** 10.3390/s120506645

**Published:** 2012-05-21

**Authors:** Fabio Rezzonico, Theo H. M. Smits, Brion Duffy

**Affiliations:** Plant Protection Division, Agroscope Changins-Wädenswil ACW, Schloss 1, Wädenswil CH-8820, Switzerland; E-Mails: fabio.rezzonico@acw.admin.ch (F.R.); theo.smits@acw.admin.ch (T.H.M.S.)

**Keywords:** LuxS, *N*-acyl homoserine lactone, *Erwinia*, *Pantoea*, *Salmonella*, *Serratia*, *Enterobacter*, metabolism, autoinducer, plant pathogen, nitrogen fixation

## Abstract

The LuxS enzyme, an S-ribosyl-homocysteine lyase, catalyzes the production of the signal precursor for autoinducer-2 mediated quorum sensing (QS-2) in *Vibrio*. Its widespread occurrence among bacteria is often considered the evidence for a universal language for interspecies communication. Presence of the *luxS* gene and production of the autoinducer-2 (AI-2) signal have repeatedly been the only evidences presented to assign a functional QS-2 to the most diverse species. In fact, LuxS has a primary metabolic role as part of the activated methyl cycle. In this review we have analyzed the distribution of QS-2 related genes in *Enterobacteriaceae* by moving the focus of the investigation from AI-2 production to the detection of potential AI-2 receptors. The latter are common in pathogens or endosymbionts of animals, but were also found in a limited number of *Enterobacteriaceae* of the genera *Enterobacter, Klebsiella*, and *Pantoea* that live in close association with plants or fungi. Although a precise function of QS-2 in these species has not been identified, they all show an endophytic or endosymbiontic lifestyle that suggests a role of type-2 quorum sensing in the adaptation to closed ecosystems.

## 1. Introduction

The historical concept of microorganisms as single entities autonomously thriving in their environment has been clearly overcome by abundant evidence demonstrating that many, if not most, bacteria are able to live in organized communities requiring the exchange of some kind of information to coordinate their behavior. Part of this communication process, known as quorum sensing (QS), is defined as the awareness of bacteria to population densities and is based on the secretion and detection of different chemical molecules that are expressed in a density-dependent manner. Several cellular mechanisms are understood to be regulated by a QS mechanism in a variety of bacteria.

Plant-associated bacteria display a multiplicity of different lifestyles ranging from the commensal epiphyte and the endophytic symbiont up to a fully pathogenic existence. Many species are able to switch between different lifestyles depending on the environmental conditions met *in planta* and alter their population structure accordingly, which may range from a free-living unicellular state to communities of organisms living within extracellular matrices known as biofilms [1]. These processes, together with swarming behavior or the production of antibiotics and virulence factors, are often steered by the QS molecules, the so-called autoinducers (AI) [2–4].

The most widespread QS system in Gram-negative bacteria (QS-1) was first discovered in the marine symbiont *Vibrio fischeri*, where it controls bioluminescence. It consists of an N-acyl-L-homoserine lactone (AHL) synthase and a transcriptional activator encoded by the genes *luxI* and *luxR*, respectively [5,6]. In the related marine bacterium *Vibrio harveyi*, AHL is synthesized by the LuxM synthase, which shows no homology to LuxI-type AHL synthases although it is based on same biochemistry. In *V. harveyi*, the signal is detected by the LuxN histidine kinase [7]. Several AHLs (or type I autoinducers, AI-1), all sharing a common homoserine lactone core, but differing in their acyl side chain moieties have been described to date [2–4]. Various sets of AI-1 molecule and cognate receptor are employed (sometimes simultaneously [8,9]) among Gram-negative bacteria. Interspecies communication is basically limited to species sharing the same system, although a limited crosstalk is possible between bacteria that use chemically similar AHLs [10–12].

**Figure 1.**
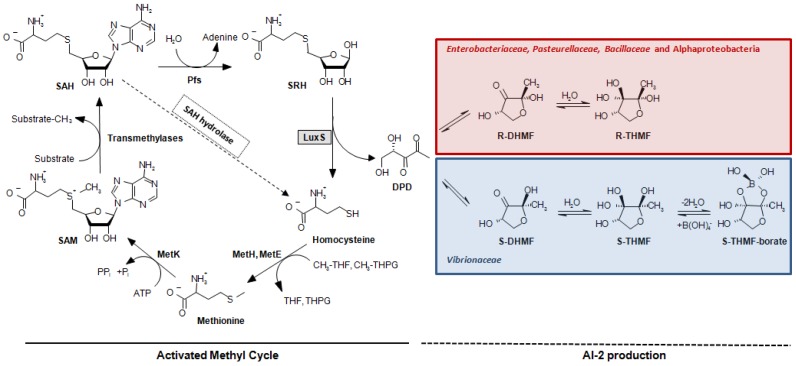
Relationship between the Activated Methyl Cycle (AMC) and AI-2 production in bacteria. The AMC is responsible for the generation of the major methyl donor in the cell, S-adenosyl-L-methionine (SAM), and the recycling of methionine by detoxification of S-adenosyl-L-homocysteine (SAH). LuxS takes part in this cycle by salvaging the homocysteine moiety from the cycle intermediate S-ribosyl-homocysteine (SRH). The alternative homocysteine-regeneration pathway using SAH hydrolase, which is employed by Alphaproteobacteria, is portrayed by the dashed arrow. As a by-product of the LuxS-catalyzed reaction, the direct AI-2 precursor 4,5-dihydroxy-2,3-pentadione (DPD) is formed. DPD undergoes further reactions to form distinct biologically active signal molecules generically termed AI-2. (2*S*,4*S*)-2-methyl-2,3,3,4-tetrahydroxytetrahydrofuryl borate (*S*-THMF-borate), the AI-2 signal of *Vibrionaceae*, is produced without the help on any known enzyme in the presence of boric acid (lower pathway), whereas in other bacteria (e.g., *S*. Typhimurium) DPD rearranges spontaneously to form (2*R*,4*S*)-2-methyl-2,3,3,4-tetrahydroxytetrahydrofuran (*R*-THMF) as AI-2 signal (upper pathway). CH_3_-THPG: *N^5^*-methyltetrahydropteroryl glutamate, CH_3_-THF: *N^5^*-methyltetrahydrofolate.

A second quorum sensing system (QS-2, [13]) was discovered in *V. harveyi* [14]. This system is controlled by the LuxS protein that catalyzes the production of (*S*)-4,5-dihydroxy-2,3-pentanedione (DPD). DPD is the direct precursor of a different autoinducer molecule (AI-2), (2*S*,4*S*)-2-methyl-2,3,3, 4-tetrahydroxytetrahydrofuryl borate (S-THMF-borate), a furanosyl borate diester [15] (Figure 1). In *Vibrio* spp., the AI-2 signal is detected by the two-component sensor kinase LuxPQ and is ultimately linked to the same transduction pathway used by the QS-1 sketched above and a third quorum sensing system, which is based on signal molecule (S)-3-hydroxytridecan-4-one (CAI-1) in *Vibrio cholerae* [16] and *Vibrio harveyi* [17]. All three signals are conveyed through the central signal relay protein LuxU and the terminal response regulator LuxO, the latter controlling gene expression together with sigma factor σ^54^ [18], whereby the strengths of the different autoinducer signals are not equivalent and vary from species to species [19,20]. In bacterial orders other than the *Vibrionaceae*, the task of detecting a different form of the DPD-derived AI-2 signal, (2*R*,4*S*)-2-methyl-2,3,3,4-tetrahydroxytetrahydrofuran (R-THMF), is carried out by an ABC-transporter, the Lsr-receptor complex. Orthologs of this protein are prevalent mainly in *Enterobacteriaceae* [21,22], *Pasteurellaceae* and *Bacillaceae*, but were not detected in *Vibrionaceae* [23]. The presence of LuxS has been reported in several subgroups of the bacterial kingdom such as Actinobacteria, Bacilli, Bacteroidetes, Deinococci and Beta-, Gamma-, and Epsilonproteobacteria. Conversely, LuxS has not been described in Archaea nor in Eukarya [24,25]. The pervasive nature of the *luxS* gene, embracing both Gram-negative and Gram-positive bacteria, led to the presumption that QS-2 may be the foundation of a bacterial Esperanto; that is to say, a universal language spoken and understood by various bacterial species [26]. Unfortunately, this assumption has often neglected a crucial function of LuxS in bacterial cells. This protein has an enzymatic role in the activated methyl cycle (AMC) [25], which accounts for the regeneration of the major methyl donor S-adenosyl-L-methionine (SAM) and the recycling of methionine by detoxification of S-adenosyl-L-homocysteine (SAH) in the cell (Figure 1). This omission has repeatedly led to the misinterpretation as QS of metabolic effects in *in vitro* mutational experiments and to the incorrect interpretation of *in silico* genomic data (see Section 5) [23]. Further complicating data interpretation, also the presence of sequences encoding accessory elements like small regulatory RNAs (sRNAs) in the coding or the promoter region of the *luxS* gene has been shown be involved in QS-related behaviors. Thus, their involvement should carefully be considered when evaluating the phenotype of *luxS* mutants [27,28].

In this review we will critically review the latest publications on QS-2 to analyze whether LuxS is involved in signaling or if it may hold a mere metabolic role in the species studied. Furthermore, we will comb through the published genomes of bacteria and search for elements related to AI-2 signaling that may allow the formulation of bioinformatics-informed predictions on the importance of QS-2, with particular focus on plant-associated *Enterobacteriaceae*.

## 2. True, Functional AI-2 Quorum Sensing Systems

### 2.1. Vibrionaceae

**Figure 2.**
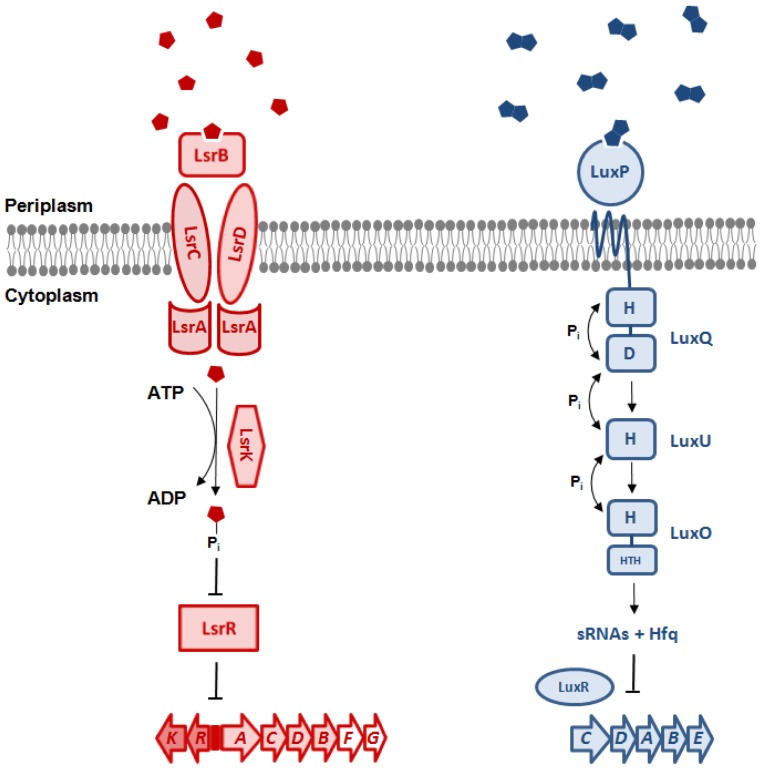
Transduction of the AI-2 signal and autoinducer gene regulation in *Enterobacteriaceae* (left, in **red**) and *Vibrionaceae* (right, in **blue**). In the *Enterobacteriaceae*, the AI-2 signal *R*-THMF is imported by the means of the Lsr ABC transporter in the cytoplasm of the cell, where is phosphorylated by LsrK. AI-2-P binds the repressor LsrR which is then released from the *lsr* promoter to allow the expression of the autoinducer operon. The histidine/aspartate (H/D) hybrid kinase LuxQ autophosporylates in the absence of autoinducers in *Vibrionaceae*. The phosporylation signal is transmitted to the histidine phosphorelay LuxU that conveys it to the σ^54^-dependent response regulator LuxO, which activates the expression of small regulatory RNAs (sRNAs). The complexes of these sRNAs and chaperone protein Hfq destabilizes the mRNA of master regulator LuxR, thereby repressing the transcription of the *lux* operon. In the presence of *S*-THMF-borate, the AI- 2 receptor LuxP converts LuxQ from kinase to phosphatase, reversing the flow of phosphate through the pathway and hence allowing the expression of the *lux* operon.

The genus *Vibrio* contains over 50 species that can be found either free-living or in association as commensals, symbionts or pathogens with fauna and flora of aquatic habitats, depending on the species [29]. In this genus, AI-2 regulated QS controls several biological functions, such as bioluminescence in the marine Gram-negative bacterium *V. harveyi*. The production of luciferase depends upon the production and detection of AI-1, AI-2 and CAI-1 in *V. harveyi*. The QS circuit consists of three parallel sensory systems [17,30]. AI-1 synthase LuxLM produces *N*-(3-hydroxybutanoyl)-L-homoserine lactone [5,14], CAI-1 is produced by the CqsA enzyme, whereas S-THMF-borate (AI-2) is synthesized by LuxS [13–15,31]. The hybrid kinase LuxQ autophosporylates at Asp-47 in the absence of the AI-2 molecule [32]. The phosphorylation signal is conveyed to the response regulator protein LuxO, which, in conjunction with alternative sigma factor σ^54^, activates the transcription of five sRNAs [33] that, in complex with the sRNA chaperone Hfq, destabilize the transcript of master regulator LuxR, repressing the transcription of the *lux* operon. At high cell density, the cognate sensors LuxN and LuxP bind AI-1 and AI-2 in the periplasm, respectively [14,34,35]. The AI-2/LuxP complex interacts with LuxQ and transduces the AI-2 signal inside the cell [15,34] by changing the activity of the latter from kinase to phosphatase [35]. This reverses the flow of phosphate through the pathway and allows the expression of the *luxCDABE* operon, that controls the production of luciferase and the emission of light by the bacteria [36] (Figure 2).

As one autoinducer is, in principle, sufficient to control density-dependent gene regulation, it was hypothesized that AI-1 and AI-2 are used by *V. harveyi* for intra- and interspecies communication, respectively. This setup makes it possible for the bacterium to distinguish between mono- and mixed-culture situations [30,37], as different combinations of autoinducers would reflect the composition and abundance of species within the microbial community (at least within the *Vibrionaceae*, which is the sole bacterial family so far known to hold the LuxPQ-type of receptors [23,38]). In addition to bioluminescence [14,34,39], metalloprotease [30], siderophore, and exopolysaccharide production [40] are also positively regulated by AI-2 in *V. harveyi* , whereas type III secretion is negatively regulated [39].

The QS mechanisms in different *Vibrio* spp. diverge in their composition and genetic response although the basic machinery is quite conserved. QS-2 is a crucial regulator of virulence in the human pathogen *V. cholerae* [19] and of the expression of colonization genes in the symbiont of the Hawaiian bobtail squid (*Euprymna scolopes*), *V. fischeri* [41,42].

Other genes whose expression is regulated by QS-2 are a metalloprotease and several virulence genes in the human and eel pathogen *Vibrio vulnificus* [43,44] or type III secretion system genes in the gastroenteritis agent *Vibrio parahaemolyticus* [39]. QS-2 is present also in the opportunistic fish pathogen *Vibrio anguillarum* [45], even if a clear role for its function could not be defined in the latter. In all these species, bioinformatics analyses detected a minimal set of QS genes (*luxS, luxPQ, luxU, luxO*) involved in the AI-2 transduction pathway [23]. Other species, such as the marine bacterium *V. angustum*, are devoid of the LuxPQ receptor and do not respond to the AI-2 signal [46].

### 2.2. Enterobacteriaceae, Pasteurellaceae, Bacillaceae and Alphaproteobacteria

Outside the *Vibrionaceae* family, another type of receptor, named Lsr (LuxS-regulated), is responsible for sensing the AI-2 signal. In *S. enterica* subsp. *enterica* serovar Typhimurium, a boron-free variant of AI-2 binds to the periplasmic LsrB protein [47], the receptor component of the AI-2 ABC transporter (Transporter Classification Database number, TC# 3.A.1.2.8, [48]). The uptake system also contains the LsrC and LsrD proteins, which form a heterodimeric membrane channel for AI-2 uptake, and LsrA, the ATPase that provides energy for AI-2 transport. Expression of the *lsrACDBFGE* operon is regulated by the AI-2 kinase LsrK as well as by the repressor LsrR, which is active in the absence of phosphorylated AI-2 (AI-2-P) (Figure 2) and was shown to specifically bind the intergenic region between *lsrR* and the *lsr* operon *in vivo* [49]. A BlastP search using LuxP of *V. harveyi* results in local hits within LsrB of *Enterobacteriaceae*. The similarity is limited to a small portion at the C-terminus of the LuxP protein, which resembles the receptor (RbsB) of the ribose ABC transporter (TC# 3.A.1.2.1). This similarity is most probably indicative of the AI-2 substrate-binding domain of both receptors. In contrast to what occurs in *Vibrionaceae*, where the AI-2 signal but not the AI-2 molecule is transduced inside the cytoplasm, this mechanism requires the import of the molecule inside the cell, where it is phosporylated by LsrK [22], thus effectively depleting AI-2 from the culture medium when the receptor is active.

Bioinformatic analyses [23,50] have demonstrated that, among LuxS positive species, the Lsr-receptor complex is generally more widely spread than the LuxPQ-system (Table 1). It was found in members of the bacterial families *Enterobacteriaceae* (genera *Escherichia, Enterobacter, Klebsiella, Yersinia, Shigella, Photorhabdus*, and *Pantoea*, but not *Serratia, Sodalis glossinidius*, or *Erwinia*), *Pasteurellaceae* (in *Actinobacillus actimycetemcomitans, Pasteurella multocida, Haemophilus somnus* and *Haemophilus influenzae*, but not in related species such as *Actinobacillus pleuropneumoniae*) or *Bacillaceae* (*Bacillus cereus* group). This type of receptor was also found on the pSymB-plasmid of *Sinorhizobium meliloti* and in the small, fast-evolving chromosomes of *Rhodobacter sphaeroides* 2.4.1 and *Rhodobacter capsulatus*, which are Alphaproteobacteria that lack the *luxS* gene and use a one-step reaction catalyzed by SAH hydrolase to detoxify SAH and regenerate homocysteine (Figure 1).

Pereira and coworkers [50] identified critical factors for predicting which LsrB orthologs are functional AI-2 receptors by assaying the AI-2 binding ability of selected candidates. Bacteria having a protein showing more than 63% amino acid sequence identity to *S.* Typhimurium LsrB, including conservation of all six AI-2 binding-site residues (K35, D116, D166, Q167, P220, and A222), were shown to have functional LsrB-like AI-2 receptors and orthologs to all of the proteins encoded by the *lsr* operon.

A second group with lower sequence identity (<36%) that contained substitutions at residues D166 and A222, did not have the complete set of orthologs for the other genes of the *lsr* operon. In these species, LsrB was shown to be likely involved in rhamnose uptake. AI-2 taken up by the Lsr system does not appear to be used as a carbon source, because both cultures of *S.* Typhimurium and *S. meliloti* are unable to grow when AI-2 is used as the sole carbon source [21,51].

In enterohemorrhagic *E. coli*, AI-2 was shown to influence chemotaxis and motility. AI-2 was suggested to be an important signal in colonization and successful infection of the human gastrointestinal tract [52,53]. Both LsrR and LsrK serve as global regulators for the expression of several important genes, those associated with host invasion and biofilm architecture being the most affected. Deletion of *lsrR* and *lsrK* influenced sRNA expression, which implies that further genes are regulated indirectly by the QS mechanism via these small riboregulators [54]. Both LsrB and L-serine chemoreceptor Tsr, but not AI-2 uptake in the cytoplasm, are necessary for sensing AI-2, suggesting that LsrB and Tsr interact directly in the periplasm [55].

In the human oral pathogen *Aggregatibacter actinomycetemcomitans*, AI-2 is internalized via the Lsr system and LsrB is required to mediate the complete AI-2-dependent activation of biofilm formation [56,57]. AI-2 can also interact specifically with RbsB, the receptor of the ribose ABC-transporter encoded by the *rbs*-operon. In this species, iron uptake and leukotoxin production were shown to be regulated by LuxS-dependent signaling [58]. In the insect pathogen *Photorhabdus luminescens*, the transcription of the *lsr* operon is induced by AI-2, which is also involved in the regulation of biofilm formation and motility [59]. Addition of AI-2 has been associated with regulation of the growth rate in *Bacillus anthracis* [60] and was shown to negatively affect biofilm formation in *B. cereus* [61], but definitive experimental evidence that the uptake of AI-2 occurs by the means of LrsB is missing.

## 3. Alternative Receptors and QS-2 Regulators

In those cases when the chemical complementation of *luxS* mutation by the means of exogenous AI-2 is successful even in the absence of genes coding for either LuxPQ or the Lsr-receptor complex, the presence of alternative receptors for AI-2 must be postulated; otherwise a sheer metabolic role of LuxS in the associated bacteria must be considered [23]. Different studies have hypothesized a number of such QS-2 related regulators and transporters.

### 3.1. Alternative Receptors

Because AI-2 is derived from S-ribosyl-homocysteine (SRH), a molecule containing a ribose moiety, and because the periplasmic AI-2-binding proteins of both *V. harveyi* and *S.* Typhimurium show at least partial sequence identity to the *E. coli* RbsB ribose-binding protein, it was speculated that the latter may also serve as an AI-2 receptor [62]. This was then demonstrated in *A. actinomycetemcomitans* [63], in which both the LsrB and RbsB proteins were shown to interact with the AI-2 molecule [56]. Their differential affinity to the AI-2 signal, depending on whether it is found as R-THMF (like in *Enterobacteriaceae*) or as S-THMF-borate (like in *Vibrionaceae*), led to the hypothesis of the synergetic use of the two receptors as borate scavangers [57]. RbsB was also found to mediate AI-2 uptake in *H. influenzae*, influencing biofilm formation and bacterial persistence in the chinchilla middle ear [64]. Although bioinformatic analysis has shown that the *lsr*-operon is present in *H. influenzae* [50], it is not known whether LsrB plays a role in AI-2 uptake in this species. The role of RbsB in AI-2 uptake has not yet been extensively investigated, but because Rbs-transporters are quite widespread among bacteria (Table 1), it is possible that RbsB may account for AI-2 internalization in a number of species, even those lacking LuxS or LsrB [23].

Another protein involved in AI-2 sensing, TlpB, which shares no sequence similarity with the previously identified AI-2 binding proteins, has been recently described in *Helicobacter pylori*, in which the AI-2 signal was shown to function as chemorepellent. TlpB is involved in a signaling pathway downstream of LuxS and upstream of the CheA histidine kinase sensor and the two CheY response regulators required for chemotaxis, which is necessary for *H. pylori* ability to colonize the gastric mucosa of rodents [65].

In *Streptococcus mutans*, 59 genes regulated by AI-2 were identified by global transcriptome analysis. The product of one of them, SMU_408, a putative permease of the NAT/NCS2 transporter family, was hypothesized to be responsible for the uptake for AI-2 [66]. Orthologs to this putative receptor are present in all *Streptococcus* spp. for which a QS-2-related phenotype has been described [23], but also in species belonging other genera, including many for which a conventional receptor for AI-2 is already known, such as *Vibrio, Bacillus* or *Pasteurella*.

### 3.2. QS-2 Regulators

In *E. coli*, initial AI-2 internalization appears to rely upon a functional phosphoenolpyruvate phosphotransferase system (PTS), a mechanism for the translocation and phosphorylation of several carbohydrates such as glucose, fructose, mannose, hexitols, and 3-glucosides [67,68]. AI-2 is first imported in a PTS-dependent manner, and LsrK, not Enzyme I of the PTS (TC# 8.A.7.1.1), is necessary for phosphorylation. AI-2-phosphate then inactivates LsrR and induces synthesis of the Lsr transporter [68]. Another potential QS-2 regulator (MqsR) was detected in *E. coli* strain K12, where it was shown to induce AI-2 dependent biofilm formation and modify biofim architecture by stimulating flagellar motion and motility [69] via response regulator QseB and sensor kinase QseC [70]. Later it was shown that *mqsR* and *ygiT* code for a new toxin-antitoxin system, represented in several groups of bacteria [71], that causes biofilm formation-associated cell death [72] via an mRNA interferase mechanism [73].

In the same strain, an YdgG null mutant showed lowered extracellular and higher intracellular concentrations of AI-2, which resulted in augmented transcription of flagellar genes leading to increased cell motility and decreased biofilm formation. Hence, it was concluded that YdgG (renamed TqsA for ‘transport of quorum-sensing signal’) controls export of AI-2 [74].

In *A. actinomycetemcomitans*, there are indications that the ArcB sensor/kinase may contribute to the signal transduction cascade that directs the LuxS-dependent expression of iron acquisition genes under conditions of iron limitation. The *arcB* mutant gives rise to a phenotype that is similar to that of a LuxS null strain [75]. The AI-2 signaling system also regulates capsular polysaccharide production in the major nosocomial pathogen *Staphylococcus aureus* through a two-component system, which was first characterized in *E. coli*. This system consists of a sensor histidine kinase KdpD that phosphorylates the response regulator KdpE, which in turn binds to the *cap* promoter [76]. The exact mechanism by which LuxS/AI-2 interacts with KdpDE is still unknown. The ability to remove AI-2 from culture supernatants was previously demonstrated in another *Staphylococcus* species (*i.e., S. epidermidis*), but the actual receptor was not identified in that study [77].

## 4. Lifestyle and Host Specificity of QS-2 Positive Bacteria

Although the two-component sensor kinase LuxPQ is expected to be limited to marine bacteria of the genus *Vibrio*, there are indications that Lsr-mediated AI-2 sensing is a trait generally restricted to (but not a prerequisite for) bacteria that live in close association with specific groups of eukaryotes. In keeping with this idea, the *lsr*-locus is present in many species of the *B. cereus* group, but not in *B. subtilis*, a soil organism which is not considered to be an animal pathogen [23]. Among *Salmonella* spp., the *lsr* operon is only located in *S. enterica* subsp. *enterica* serovars that are associated with endotherms, whereas it is absent from the genome of *S. bongori*, a species usually associated with cold-blooded animals [78]. In the nematode symbiont *P. luminescens* the Lsr-receptor complex is apparently restricted to those strains that form a mutualistic consortium with *Heterorhabditis bacteriophora*, but the *lsr* genes show extensive deletions in strains associated with other hosts, suggesting that QS-2 is deeply involved in the association with this particular insect-pathogenic nematode [79]. Thus, the pattern of distribution of LsrB among the different bacterial species suggests that AI-2 may be a relevant signal for many animal pathogens and symbionts and that the Lsr-receptor complex may play a general role in host colonization by regulating processes, such as biofilm formation, for which a high cellular density is a critical prerequisite.

**Figure 3.**
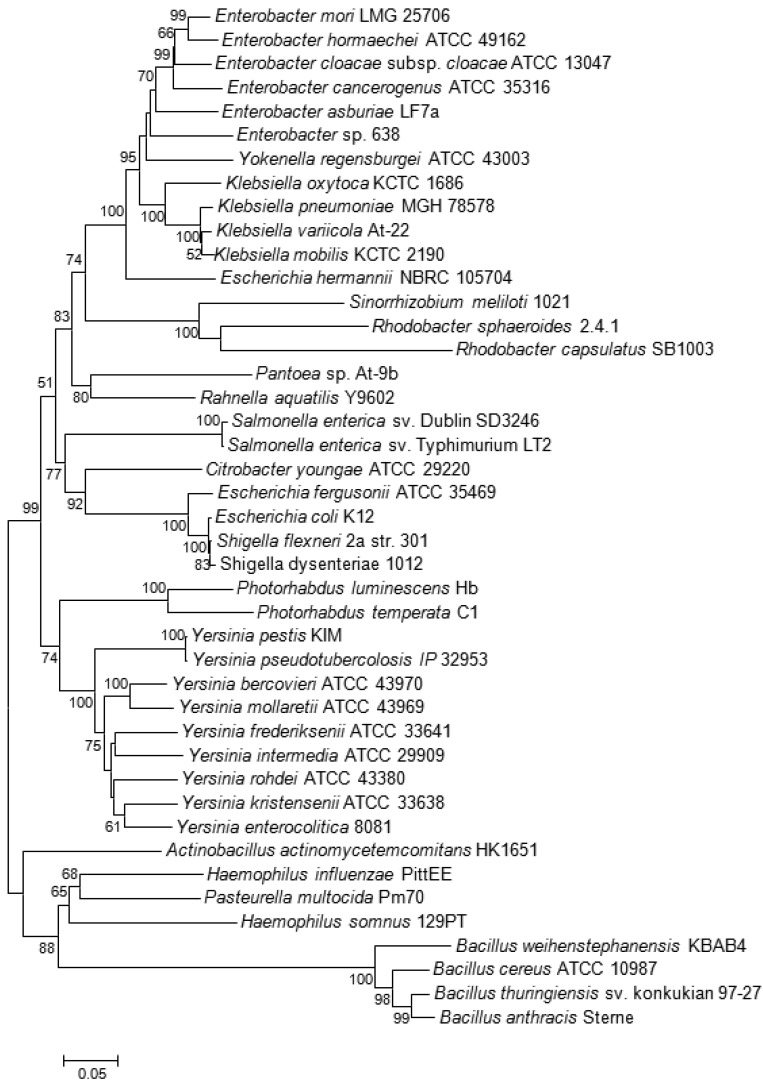
Phylogeny of *lsrB* positive bacteria based on complete *lsrB* sequences. The distance tree was generated by the NJ method with the JC formula, without choosing any outgroup. Nodal supports were assessed by 1000 bootstrap replicates. Only bootstrap values greater than 50% are shown. All *lsrB* sequences were retrieved from published genome projects at the NCBI database.

Among plant-associated bacteria, AI-2 specific receptors were only found on the pSymB-plasmid of the N_2_-fixing Alphaproteobacterium *S. meliloti*, which forms an endosymbiotic relationship with legumes from the genera *Medicago, Melilotus* and *Trigonella*. Both *S. meliloti* (as well as related Alphaproteobacteria *R. sphaeroides* and *R capsulatus*) and the aforementioned *Bacillus cereus* group are thought to have acquired the genes coding for the Lsr receptor complex by horizontal gene transfer (HGT), probably from bacteria belonging to the *Enterobacteriaceae* and *Pasteurellaceae*, respectively, that share the same ecological niche [23,50]. This conclusion is supported by the unusual position of their *lsrB* genes in the corresponding phylogenetic tree (Figure 3). As Alphaproteobacteria themselves are unable to produce AI-2, it was speculated that they may use the Lsr-receptor complex to eavesdrop on the AI-2-mediated communication of other bacterial species in the same habitat [23,51].

*Klebsiella* spp. naturally occur as a free-living diazotrophs in soil or as endophytes in the roots of a wide variety of plant species. About 30% of strains can fix nitrogen under anaerobic conditions, but isolates that are close relatives of these diazotrophs are also associated with human diseases [80,81]. Isolates forming a clade that includes the type strain of *Klebsiella variicola* were also found within fungus gardens of the colonies leaf-cutter ants of the genera *Atta* and *Acromyrmex* where, together with bacteria belonging to the genus *Pantoea*, they participate in nitrogen fixation for the fungal symbiont [82]. Both bacterial species living within this consortium carry, beside the *nif* genes coding for the nitrogen fixation pathway, the *lsr*-operon needed for the AI-2 ABC transporter. Although prevalent in the genomes of different *Klebsiella* spp., none of the two gene clusters was previously reported in any sequenced *Pantoea* spp. [83–88] suggesting that these were acquired by *Pantoea* sp. At-9b through HGT during adaptation to this particular ecological niche [82]. However, the *lsr*-genes were not directly acquired from *Klebsiella*, as the significant distance between the two genera in the *lsrB* tree demonstrates (Figure 3). Just as in the symbiotic/pathogenic interactions involving gut colonization of insects and animals, the lifestyle needed for nitrogen fixation in root nodules or in fungus gardens entails high bacterial population densities within a closed system, thus providing a possible rationale for the presence of a QS system. A potential relationship between QS-2 and nitrogen fixation is confirmed by the presence of the Lsr receptor complex in another Alphaproteobacterium *R. sphaeroides* 2.4.1. This bacterium is similar to *Sinorhizobium* in that it does not carry the *luxS* gene, but, in contrast to the latter, it is a free-living diazotroph.

In plant-associated bacteria that are not N_2_-fixing symbionts, the genes encoding the Lsr receptor complex could be identified only in the genomes of the mulberry-pathogen *Enterobacter mori* [89], in the plant growth-promoting endophyte *Enterobacter* sp. 638 [90], and in *Enterobacter cancerogenus*, which was originally isolated from poplars (*Populus* spp.) affected by a canker disease [91]. *E. cancerogenus* has also been found as clinical isolate [92]. Whether the AI-2 receptor plays a role in pathogenicity or plant association is unknown, but its presence could again reflect the importance of QS-2 in situations associated with bacterial populations thriving in a limited space such as in the case of bacterial cancers or in bacteria employing an endophytic lifestyle.

## 5. Pitfalls

The major pitfall when analyzing the role of QS-2 in a microbial species has been the reliance on the mere presence of the *luxS* gene in the genome of the bacteria under investigation [23]. As the AI-2 precursor DPD is normally produced as a by-product of the AMC, even detection of AI-2 molecules in the culture supernatant, either by chemical analysis or through reporter strains such as *V. harveyi* BB170 [31], cannot be considered as an unmistakable proof of the existence of a functional QS-2 system. The same applies to traditional mutational analysis that, given the important role of LuxS in methionine recycling within the cell, is not able to discriminate between QS-2 and metabolism-related changes of the *luxS* mutant phenotype. This is because conventional *in trans* complementation will overcome defects induced in either pathway. Examples for this kind of “missed approach” are available in a wide variety of *luxS* positive bacteria [23], including plant pathogens such as *Pectobacterium carotovorum* [93,94] or *Erwinia amylovora* [95]. In the latter study, the effects of the *luxS* mutation were sweepingly ascribed to QS without considering any possible implication of the AMC, even though it was previously demonstrated that the mutant strain has impaired metabolic fitness, especially under sulfur-limiting conditions [96]. In the same vein, the failure to complement the phenotype of a Δ*luxS* variant by the *luxS*-positive wild-type strain in co-cultivation assays suggested the absence of an extracellular signal. A way to circumvent the complication of the dual role of LuxS in the cell is to shift the focus of the analysis from LuxS itself to the analysis of potential AI-2 receptors and the chemical complementation of the *luxS* mutant, including objective evidence of AI-2 binding or uptake [97,98]. Alternatively, it was proposed to replace the *luxS* gene not with an intact copy of itself, but with the *sahH* gene that encodes the SAH hydrolase, which is part of the one-step S-adenosylhomocysteine detoxification pathway (Figure 1). In this manner, it is possible to repair the function of the AMC without restoring AI-2 production: if the wild-type phenotype can be salvaged by an *in trans* complementation with *sahH*, then any difference observed in the *luxS* mutant has to be ascribed to metabolism, not to QS-2 [99]. Conversely, if it possible to fully complement the *luxS* mutant by adding exogenous AI-2 to the culture medium (chemical complementation), a role of the latter molecule in signaling must be considered.

A more promising tactic to determine whether QS-2 is really present in a bacterial species is to shift the focus of the investigation from AI-2 production to AI-2 recognition and detection. This may include bioinformatics approaches to scan for orthologs of known AI-2 receptors [23,38,50], which must withstand the reciprocal best-hit strategy to distinguish between orthologous and paralogous genes [100]. In this kind of approach, it is important that the actual receptor is employed in similarity searches, as the use of associated proteins or domains (e.g., transmembrane channel LsrCD or sensor kinase/phosphatase LuxQ) may yield multiple partial positive matches in several species, as these proteins may share an high degree of sequence identity among paralogous systems. A thorough analysis of the complete genomes of *E. amylovora* [101] and the closely related species *E. pyrifoliae* [102], *E. piriflorinigrans* [103], *E. billingiae* and *E. tasmaniensis* [104] revealed the complete absence of any specific receptor associated with QS-2, thus confirming the metabolic role of LuxS in the fully sequenced species of this genus. In addition to the bioinformatics approach, experimental evidence of a functional QS-2 system can be gathered by confirming AI-2 depletion from the culture media [97,98] or, as more recently proposed, the use of a DPD-based rhodamine-conjugated dendrimer as multivalent probe that allows specific imaging of cells bearing Lsr-type quorum-sensing receptors [105].

## 6. Reflections

The fact that many of the alternative AI-2 receptors and QS-2 regulators are fairly common across different bacterial taxa and are not specific to *luxS*-positive bacteria suggests that they were recruited subsequently into their role in QS-2. This raises the question of whether their primary function is always true signalling or, especially in *luxS*-negative bacteria, whether it may also concern other types of interactions involving metabolism or activation of non-QS-2 related responses. In principle, because the PTS system initiates AI-2 import in *E. coli* [68] is virtually ubiquitous within the bacterial kingdom [106], almost every bacterium should at least theoretically be capable to internalize AI-2. The same is true for ribose ABC transporters, which were shown to be able to mediate AI-2 intake because of the structural similarity of the autoinducer molecule with their cognate substrate [56,63]. This could explain the successful chemical complementation of *luxS* mutants in those species that were found to be devoid of both major AI-2 receptors [23]. However, whether the transcriptional changes induced by the addition of exogenous AI-2 in the receiver organism are effectively related to QS-2 remains to be demonstrated. From an evolutionary point of view, for a secreted molecule to be a signal between different species, it must not only elicit a response in the receiving organism but also have evolved primarily to do so [107]. Interactions involving other types of non-concerted responses may be referred to as cues or coercion events [108]. A cue describes an emission which provides beneficial information to a receiver but which did not evolve explicitly for that purpose, and thus does not directly benefit the emitter. Conversely, coercion describes the situation in which a signal induces a detrimental response in a receiver to the benefit of the emitter [109]. Whatever names are used for these alternative activities, they illustrate the danger of concluding that a single prevailing environmental factor controls bacterial behavior in a manner that evolved specifically to carry out that function. In reality, both the ecological context of QS regulation and the process itself are complex and respond to a multiplicity of environmental stimuli. For this reason, the notion of ‘quorum sensing’ should perhaps be defined more narrowly to include only signals specifically used for communication between emitter and receiver cells. Otherwise, the concept of QS may be diluted to include environmental signals that affect bacterial metabolism and behavior in rather indirect ways that are not examples of intentional cell-to-cell- communication [110].

## Figures and Tables

**Table 1.** Presence of AI-2 receptors and regulators in different genera belonging to the Enterobacteriaceae or other bacterial families ^a^.

**Protein** ^b^	LuxP *V. harveyi* AAA20837	LsrB *S. enterica* Q8Z2X8	RbsB *E. coli* BAE77537	PTS *E. coli* BAA16290	MqsR *E. coli* YP_001731885	TlpB *H. pylori* YP_003056929	SMU_408 *S. mutans* NP_720856
**Genera of the *Enterobacteriaceae* family**							
*Brenneria* ^c^	-	-	+	+	-	-	(±)
*Citrobacter*	-	+	+	+	-	-	(±)
Lsr-positive species: *C. youngae*							
*Cronobacter*	-	-	+	+	-	-	(±)
*Dickeya*	-	-	+	+	-	-	(±)
*Enterobacter*	-	+	+	+	-	-	(±)
Lsr-positive species: *E. asburiae, E. cancerogenus, E. cloacae, E. hormachei, E. mori, Enterobacter* sp. 638							
*Erwinia*	-	-	+	+	-	-	(±)
*Escherichia*		+	+	+	+	-	(±)
Lsr-positive species: *E. coli, E. fergusonii, E. hermanii*							
*Klebsiella*	-	+	+	+	-	-	(±)
Lsr-positive species: *K. mobilis* (*Enterobacter aerogenes*), *K. oxytoca, K. pneumonia, K. variicola*							
*Pectobacterium*	-	-	+	+	-	-	(±)
*Pantoea*	-	+^d^	+	+	-	-	(±)
Lsr-positive strain: *Pantoea* sp. At-9b							
*Photorhabdus*	-	+	+	+	-	-	(±)
Lsr-positive species: *P. luminescens, P. temperata*							
*Rahnella* ^e^	-	+	+	+	+	-	(±)
Lsr-positive strains: *R. aquatilis* CIP 78.65, *Rahnella* sp.Y9602							
*Salmonella*	-	+	+	+	-	-	(±)
Lsr-positive species: *S. enterica* subsp. *enterica*							
*Serratia*	-	-	+	+	-	-	(±)
*Shigella*	-	+	+	+	-	(-) ^f^	(±)
Lsr-positive species: *S. flexneri, S. dysenteriae*							
*Yersinia*	-	+	+	+	+	-	(±)
Lsr-positive species: *Y. bercovieri, Y. fredericksenii, Y. enterocolitica,Y. intermedia, Y. kristensenii, Y. mollaretii, Y. pestis, Y. pseudotubercolosis, Y. rohdei*							
*Yokenella* ^g^	-	+	+	+	-	-	(±)
Lsr-positive strain: *Y. regensburgei* ATCC 43003							
**Genera belonging to other bacterial families**							
*Actinobacillus*	-	+	+	+	-	-	(±)
Lsr-positive species: *A. actinomycetmcomitans*							
*Bacillus*	-	+	+	+	-	(-) ^f^	(±)
Lsr-positive species: *B. anthracis, B. cereus, B. thuringensis, B. weihenstephanensis*							
*Burkholderia*	-	-	(±) ^h^	(±)	+	-	(±)
*Haemophilus*	-	+	+	+	-	-	(±)
Lsr-positive species: *H. influenzae, H. somnus*							
*Helicobacter*	-	-	-	(-) ^i^	-	+	(±)
*Pasteurella*	-	+	-	+	-	-	(±)
Lsr-positive species: *P. multocida*							
*Pseudomonas*	-	-	(±) ^h^	(±)	(+)	-	(±)
*Rhodobacter*	-	+	(±)	(±)	-	-	-
Lsr-positive strains: *R. sphaeroides* 2.4.1, *R. capsulatus* SB1003							
*Sinorhizobium*	-	+	(±)	(±)	-	-	(±)
Lsr-positive strain: *S. meliloti* Rm1021							
*Staphylococcus*	-	-	+	(+)	-	-	(±)
*Streptococcus*	-	-	(±) ^h^	(+)	-	-	+
*Xanthomonas*	-	-	-	(±)	-	-	(±)
*Xylella*	-	-	-	(±)	(+)	-	-
*Vibrio*	+	-	+	+	-	(-) ^f^	(±)

a Symbols represent the sequence identity with respect to the protein reference sequence used for BlastP analysis: +:sequence identity >60%; (+): sequence identity 50–60%; (±): sequence identity 30–50%; (-): sequence identity <30%; -: no relevant sequence identity;

b Organism of origin and accession number of the NCBI entry used for BlastP analysis are indicated below the protein name and and colored in red in the table;

c Only one complete genome available (*Brenneria* sp. EniD312);

d Only one *lsr*-positive sequence found: *Pantoea* sp. At-9b (YP_004116560);

e Only two complete genomes available, both *lsr*-positive: *Rahnella aquatilis* CIP 78.65 (AEX52447); *Rahnella* sp. Y9602 (ADW74190);

f annotated as chemotaxis receptor (CheW domain). CheW-like proteins are widespread among bacteria and are present in a majority of *Enterobacteriaceae*, but similarity between these bacteria and *H. pylori* is very low;

g Only one complete genome available: *Yokenella regensburgei* ATCC 43003 (EHM49552);

h annotated as RbsB despite low sequence identity with *E. coli* RbsB;

i annotated as PTS component despite low sequence identity with *E. coli* PTS.
